# Supplementary material for: Ab Initio Simulation of the Ultrafast Circular Dichroism Spectrum of Provitamin D Ring-Opening
Source: J Phys Chem Lett. 2023 May 25;14(21):5061–8. doi: 10.1021/acs.jpclett.3c00862 (PMC10240533; doi:10.1021/acs.jpclett.3c00862)
Supplement: Supplementary file 1 — jz3c00862_si_001.pdf [file jz3c00862_si_001.pdf]

Supporting Information: Ab Initio Simulation of the Ultrafast Circular Dichroism Spectrum of Provitamin D Ring-opening

Enrico Tapavicza,* Trevor Reutershan, and Travis Thompson

Department of Chemistry and Biochemistry, California State University, Long Beach, 1250 Bellflower Boulevard, Long Beach, CA, 90840

E-mail: enrico.tapavicza@csulb.edu

Calculation of time-resolved circular dichroism spectra

Circular dichroism (CD) spectra can be efficiently calculated by time-dependent density functional theory linear response theory.[1–3] For a detailed discussion on how to calculate *static* CD spectra and rotatory strengths using TDDFT, the reader is referred to the review article of Warnke and Furche.[3]

Here, we describe in detail how the time-resolved CD spectrum (TRCD) is computed based on the implementation of Furche et al.:[1,2]

To compute the instantaneous CD spectrum of one trajectory $\Delta\epsilon_i(\tau)$, we assume that the CD signal is caused by ground state absorption, rather than excited state absorption. Provided that the UV pump-pulse induces a $S_1 \leftarrow S_0$ transition, this is a reasonable assumption if the probe wavelength is also in the UV region, since higher $S_n \leftarrow S_1$ absorption energies usually appear at lower energies than the excitation energy of S_1. According to this assumption, a trajectory only contributes to the instantaneous CD spectrum once it has

undergone a transition to the ground state (Figure S1). For the ensemble averaged instantaneous spectrum $\Delta\epsilon(\tau)$, only the fraction of molecules that have already been relaxed to the ground state after initial excitation gives rise to the CD signal at delay time τ, according to Eq. 5 in the main article.

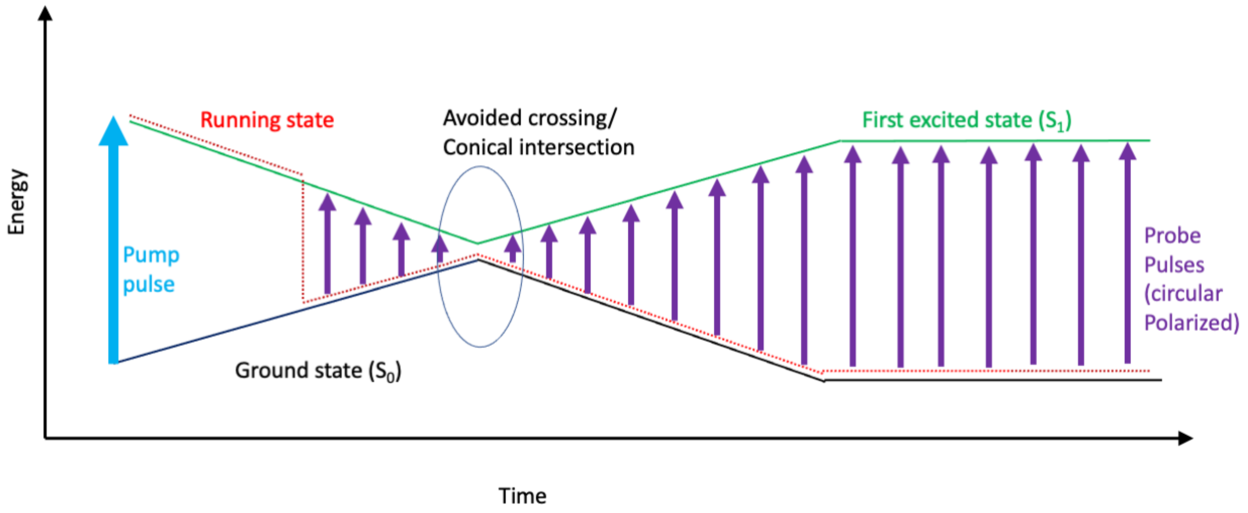

Figure S1: Scheme for the calculation of the instantaneous CD spectrum $\Delta\epsilon_i(\tau)$ for one trajectory. The instantaneous spectrum, obtained by the circular polarized probe pulses (purple), is only taken into account if the molecule has undergone a transition to the ground state.

Within surface hopping, the TRCD signal at time delay time τ is then calculated by adding the static spectrum of the parent computed, i.e. the CD spectrum at time $\Delta\epsilon_0$ to the instantaneous spectrum $\Delta\epsilon(\tau)$:

$$\Delta CD(\tau) = \Delta\epsilon_0 + \Delta\epsilon(\tau)\,. \tag{1}$$

Any excited state absorption is neglected in this method.

Analysis of high-frequency oscillations

To analyze the high-frequency, low-amplitude oscillations in the TRCD, we examine one example trajectory (Figure refdistances). The high-frequency oscillations in the TRCD spectrum is caused by high-frequency oscillations in the rotatory strength of the S_1 (panel **B**), which occurs with similar frequency as the bond vibrations in the central unit of the provitamin D/previtamin D (panel **D**). According to the dynamophore concept,[4] the oscillations in cyclohexadiene unit are activated by the electronic transition to S_1, which eventually leads to the electrocyclic ring-opening to form the hexatriene derivative previtamin D.

Another reason for oscillations in the traces of the TRCD spectrum are the oscillations in the S_1 excitation energy (panel **A**). The maxima of absorption bands is given by the S_1 excitation energy; therefore the lambda-max values of the instantaneous spectra oscillate. However, since the traces of the broadband spectrum are taken at constant wavelengths, the oscillations in the excitation energies lead to an oscillation in the TRCD traces. This oscillation occurs with similar frequency as the bond oscillations of the central unit of provitamin D/previtamin D.

In summary, the high-frequency, low-amplitude oscillations are possibly due to the density fluctuations associated to the bond vibrations in the central double bond system of the molecule.

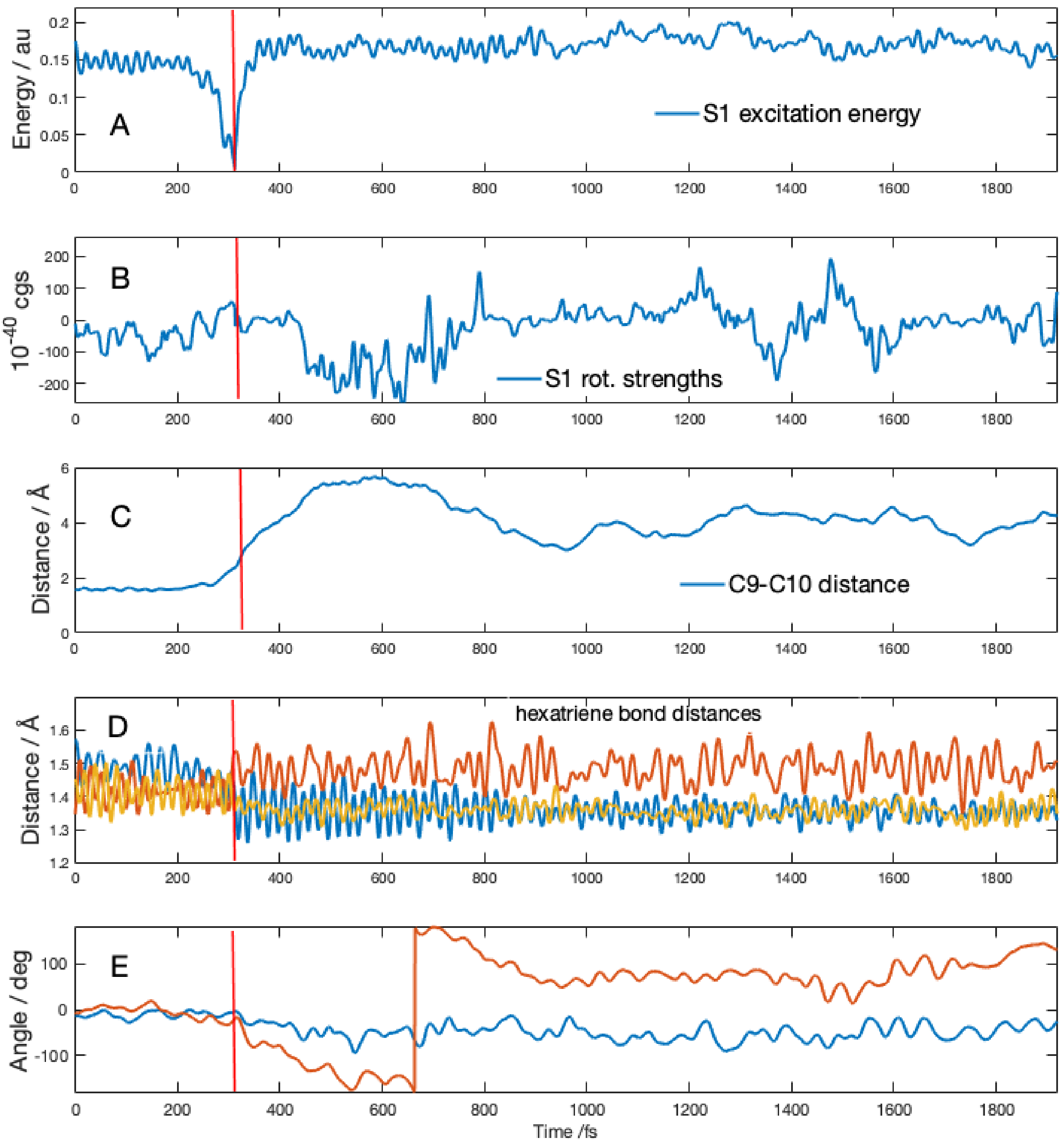

Figure S2: Time evolution of different parameters of an example ring-opening trajectory. The vertical line at 313 fs indicates the surface hop from S_1 to the ground state. **A**. Evolution of the S_1 excitation energy in au. **B**. Evolution of the rotatory strengths of S_1. **C**. Evolution of the bond-breaking coordinate (C9-C10 bond). **D**. Evolution of some of the bond distances in the hexatriene unit; blue: distance C9-C8, red: distance C8-C7, yellow: distance C7-C6. **E**. Evolution the dihedral angles ϕ_1 (red) and ϕ_2 (blue). Atom numbers are defined in Figure 1 of the main article.

References

(1) Furche, F. On the density matrix based approach to time-dependent density functional response theory. *J. Chem. Phys.* **2001**, *114*, 5982–5992.

(2) Furche, F.; Ahlrichs, R. Adiabatic time-dependent density functional methods for excited state properties. *J. Chem. Phys.* **2002**, *117*, 7433.

(3) Warnke, I.; Furche, F. Circular dichroism: electronic. *Wiley Interdiscip. Rev. Comput. Mol. Sci.* **2012**, *2*, 150–166.

(4) Schalk, O.; Geng, T.; Thompson, T.; Baluyot, N.; Thomas, R. D.; Tapavicza, E.; Hansson, T. Cyclohexadiene Revisited: A Time-Resolved Photoelectron Spectroscopy and ab Initio Study. *J. Phys. Chem. A* **2016**, *120*, 2320–2329.
