# Supplementary material for: Ab Initio Simulation of the Ultrafast Circular Dichroism Spectrum of Provitamin D Ring-Opening
Source: J Phys Chem Lett. 2023 May 25;14(21):5061–8. doi: 10.1021/acs.jpclett.3c00862 (PMC10240533; doi:10.1021/acs.jpclett.3c00862)
Supplement: Supplementary file 2 — jz3c00862_si_002.pdf [file jz3c00862_si_002.pdf]

jz-2023-008623.R1

Name: Peer Review Information for "Ab initio Simulation of the Ultrafast Circular Dichroism Spectrum of Provitamin D Ring-opening"

First Round of Reviewer Comments

Reviewer: 1

Comments to the Author

In this manuscript, Tapavicza, et al. presented a scheme to simulate ultrafast pump-probe time-resolved circular dichroism (TRCD) spectra based on mixed quantum-classical nonadiabatic dynamics method of surface hopping. This is very useful for interpreting the conformational changes in nonadiabatic excited state processes. The manuscript is well organized and the results are very interesting. I believe this work is publishable in The Journal of Physical Chemistry Letters after addressing the following minor issues:

1. Are the simulation and experiment in this work undertaken in gas phase or in solution? This should be clearly described.

2. Why did the authors choose hybrid PBE0 functional for the studied system?

Reviewer: 2

Comments to the Author

In this work, Tapavicza et. al. tried to perform the ab initio dynamics simulation of the ultrafast time-resolved circular dichroism spectrum of provitamin D ring opening reactions. The systems understand are rather interesting due to their rich photochemistry and the importance of these compounds. In addition, this work also one of the first works, at least in my knowledge, to simulate the time-resolved CD spectrum by using on-the-fly surface hopping dynamics at all atomic levels. In this sense, I think that this work is interesting while some improvements are also necessary.

1. The authors give a very short discussion on how to simulate the time-resolved CD spectrum. I can understand that this is due to the fact that the authors wish to write a short paper. However, I strongly recommend to prepare a supporting information (SI) and give a full description on all method parts in SI. This way also can provide a very detailed explanation on Eq 1-4, including all terms in these equations. Currently, some labels are not explained.

2. It seems that the authors do not use decoherent correction. This may decrease the reliability of the surface hopping dynamics. Why not include such corrections? Without such corrections, some errors may appear.

3. In Figure 2, it looks that different isomers are generated. Is it possible to correlate Figure 2 with Figure 1. For example, label the positions of each isomers in Figure 2?

4. In Figure 5, I noticed that the signals are highly oscillated and some high-frequency oscillations seem to exist. How to understand such high-frequency oscillations?

5. It looks that many isomers are generated in the ground state after the internal conversion. Is it possible to give a Scheme to illustrate the whole mechanism, explaining the generation of all products and their correlation with time-resolved CD spectrum?   This will largely help people to understand the whole mechanism.

6. The simulation of the time-resolved spectrum is a very important topics in the on-the-fly nonadiabatic surface hopping dynamics field. More additional discussions on this field should be given and some important references should be mentioned.

Author's Response to Peer Review Comments:

Reviewer: 1

> **Reviewer Comment:**
>
> *Reviewer: 1*
>
> *Recommendation: This paper is publishable subject to minor revisions noted. Further review is not needed.*
>
> *Comments: In this manuscript, Tapavicza, et al. presented a scheme to simulate ultrafast pump-probe time-resolved circular dichroism (TRCD) spectra based on mixed quantum-classical nonadiabatic dynamics method of surface hopping. This is very useful for interpreting the conformational changes in nonadiabatic excited state processes. The manuscript is well organized and the results are very interesting. I believe this work is publishable in The Journal of Physical Chemistry Letters after addressing the following minor issues:*

Authors' response: We thank the reviewer for their constructive comments.

> **Reviewer Comment:**
>
> *1. Are the simulation and experiment in this work undertaken in gas phase or in solution? This should be clearly described.*

Authors' response: We clarified in the introduction that the simulations have been carried out in the gas phase, whereas the experimental TRCD has been measured in ethanol solution:

> Here, we apply time-dependent density functional theory surface hopping (TDDFT-SH) molecular dynamics simulations to model the TRCD along the photoinduced ring-opening reaction of provitamin D in the gas phase. This reaction constitutes the initial step in natural vitamin D photosynthesis. Experimentally, the TRCD spectrum of this reaction has been measured in ethanol solution [8].

Furthermore, we point this out in the results section:

> The time in our gas phase simulations until the oscillations approximately disappear and a relatively constant TRCD signal is reached (≈ 4 ps) is much shorter than in the experimental solution phase spectrum (≈ 14 ps).

> **Reviewer Comment:**
>
> *2. Why did the authors choose hybrid PBE0 functional for the studied system?*

Authors' response: The PBE0 approximation to the exchange correlation functional has been shown to predict accurate excitation energies for a wide range of organic molecules, as the benchmark study of Send et al. has shown [11]. In our earlier study [14], PBE0 gave similar results as the correlated second-order approximate coupled cluster method (CC2). We added this information to the section **Computational Details** in the manuscript:

> The PBE0 functional has been shown to accurately predict excitation energies for a wide range of organic compounds [11]; for provitamin D, it predicts excitation energies with similar accuracy as second-order approximate coupled cluster [14].

Reviewer: 2

Reviewer Comment:

Recommendation: This paper may be publishable, but major revision is needed; I would like to be invited to review any future revision.

Comments: In this work, Tapavicza et. al. tried to perform the ab initio dynamics simulation of the ultrafast time-resolved circular dichroism spectrum of provitamin D ring opening reactions. The systems understand are rather interesting due to their rich photochemistry and the importance of these compounds. In addition, this work also one of the first works, at least in my knowledge, to simulate the time-resolved CD spectrum by using on-the-fly surface hopping dynamics at all atomic levels. In this sense, I think that this work is interesting while some improvements are also necessary.

Authors' response: We thank the reviewer for the encouraging evaluation and the critical comments, which we will address in the following.

Reviewer Comment:

1. The authors give a very short discussion on how to simulate the time-resolved CD spectrum. I can understand that this is due to the fact that the authors wish to write a short paper. However, I strongly recommend to prepare a supporting information (SI) and give a full description on all method parts in SI. This way also can provide a very detailed explanation on Eq 1-4, including all terms in these equations. Currently, some labels are not explained.

Authors' response: We corrected Eq. 2 and added the missing labels for the speed of light (c), the electric dipole moment component ($\mu^{(j)}$), and the excitation energy Ω_{0n} .

"... resulting from the solution of the time-dependent Kohn-Sham eigenvalue problem [2, 1, 5],

$$G_{jk}(z) = -\frac{c}{z}\langle \mu^{(j)}|X^{(j)}(z), Y^{(k)}(z)\rangle \,, \tag{1}$$

where c denotes the speed of light and $\mu^{(j)}$ is the electric dipole moment operator. According to

$$\begin{aligned}\mathrm{Im}[G(\omega)] \quad = \quad & \frac{c\pi}{3}\sum \frac{1}{\Omega_{0n}}(R_{0n}\delta(\omega-\Omega_{0n}) \\ & \qquad -R_{0n}\delta(\omega+\Omega_{0n}))\,,\end{aligned} \tag{2}$$

where Ω_{0n} denotes the excitation energy of state n, this quantity is related to the rotatory strength ..."

For a complete description of the calculation of *static* CD spectra, i.e. Eqs. 1-4, by linear response TDDFT, however, we refer the reader to the review article by Warnke and Furche [16]. Our article focuses on the calculation of the *time-resolved* spectra, which is based on the implementation of static spectra of Furche et al. [5, 4] in the quantum chemistry program TURBOMOLE. We added more explanations on our method on how to compute TRCD to the SI. In addition, we supported our explanation by an additional scheme in the SI:

Calculation of time-resolved circular dichroism spectra

Circular dichroism (CD) spectra can be efficiently calculated by time-dependent density functional theory linear response theory [5, 4, 17]. For a detailed discussion on how to calculate *static* CD spectra and rotatory strengths using TDDFT, the reader is referred to the review article of Warnke and Furche [17].

Here, we describe in detail how the time-resolved CD spectrum (TRCD) is computed based on the implementation of Furche et al. [5, 4]:

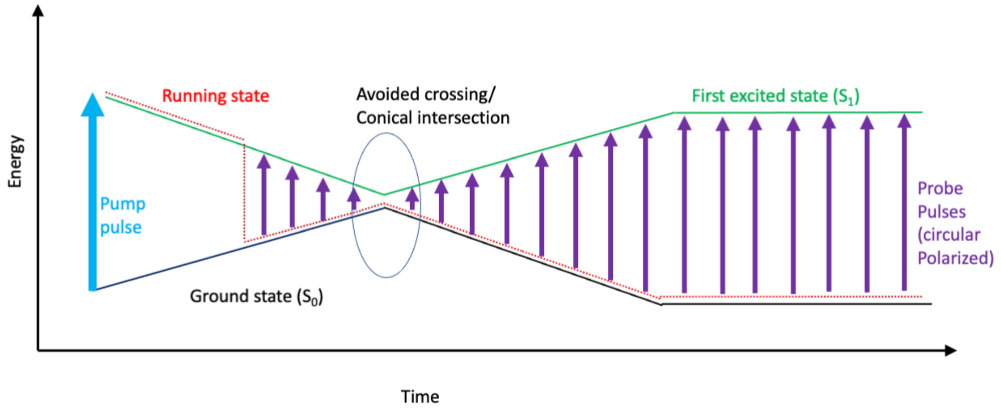

Figure 1: Scheme for the calculation of the instantaneous CD spectrum $\Delta\epsilon_i(\tau)$ for one trajectory. The instantaneous spectrum, obtained by the circular polarized probe pulses (purple), is only taken into account if the molecule has undergone a transition to the ground state.

> To compute the instantaneous CD spectrum of one trajectory $\Delta\epsilon_i(\tau)$, we assume that the CD signal is caused by ground state absorption, rather than excited state absorption. Provided that the UV pump-pulse induces a $S_1 \leftarrow S_0$ transition, this is a reasonable assumption if the probe wavelength is also in the UV region, since higher $S_n \leftarrow S_1$ absorption energies usually appear at lower energies than the excitation energy of S_1. According to this assumption, a trajectory only contributes to the instantaneous CD spectrum once it has undergone a transition to the ground state (Figure S1). For the ensemble averaged instantaneous spectrum $\Delta\epsilon(\tau)$, only the fraction of molecules that have already been relaxed to the ground state after initial excitation gives rise to the CD signal at delay time τ, according to Eq. 5 in the main article.
>
> Within surface hopping, the TRCD signal at time delay time τ is then calculated by adding the static spectrum of the parent computed, i.e. the CD spectrum at time $\Delta\epsilon_0$ to the instantaneous spectrum $\Delta\epsilon(\tau)$:
>
> $$\Delta CD(\tau) = \Delta\epsilon_0 + \Delta\epsilon(\tau)\,. \tag{3}$$
>
> Any excited state absorption is neglected in this method."

> **Reviewer Comment:**
>
> *2. It seems that the authors do not use decoherent correction. This may decrease the reliability of the surface hopping dynamics. Why not include such corrections? Without such corrections, some errors may appear.*

Authors' response: Indeed, we did not use decoherent correction. As we pointed out, our calculations are based on earlier surface hopping simulations [14] without decoherent correction. While the decoherent correction would probably lead to a better agreement in the decay time, they do not affect the general interpretation of our simulations. There are many aspects at which the accuracy of our method can be incrementally improved; we here focus on the general presentation of the method and on its application to provitamin D ring-opening.

Reviewer Comment:

3. In Figure 2, it looks that different isomers are generated. Is it possible to correlate Figure 2 with Figure 1. For example, label the positions of each isomers in Figure 2?

Authors' response: We labeled the different rotational isomers in Figure 2 according to Figure 1. We also added some labels for rotamers in Figure 4, left column.

New Figure 2:

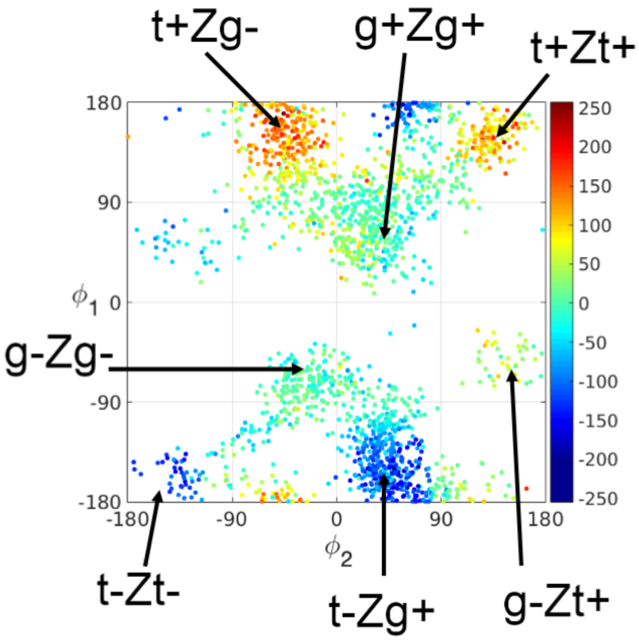

New Figure 4:

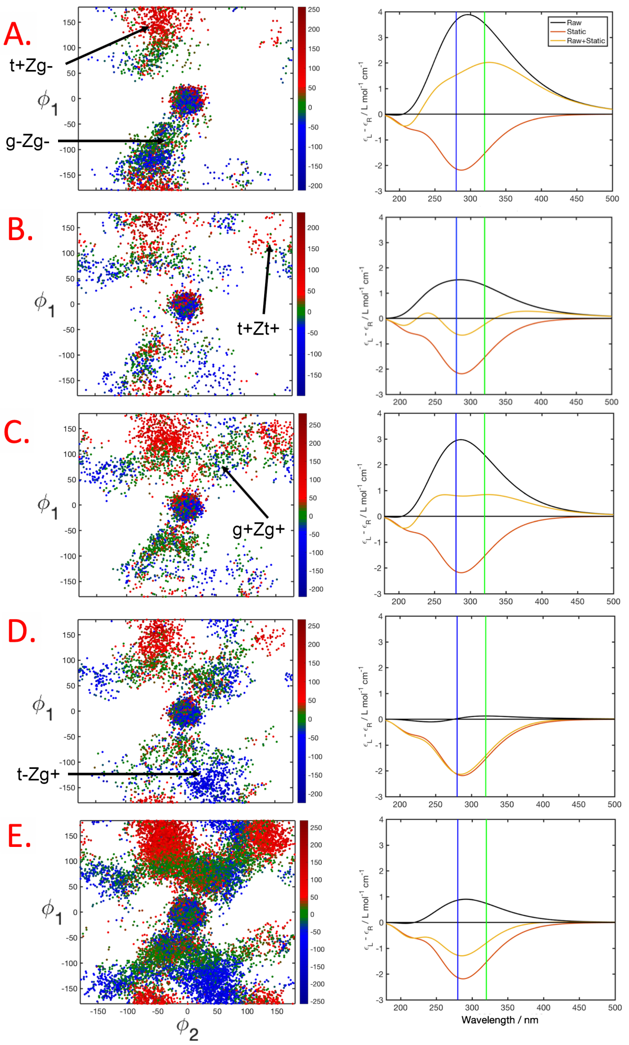

Reviewer Comment:

4. In Figure 5, I noticed that the signals are highly oscillated and some high-frequency oscillations seem to exist. How to understand such high-frequency oscillations?

Authors' response: The high frequency oscillations are due to bond length oscillations in the central conjugated double bond system of provitamin D/previtamin D. We added an additional figure of one example trajectory to the supporting information to illustrate this. Figure S1 shows that the high-frequency oscillations in the rotatory strengths (panel **B**) that are superimposed to the large-amplitude variations occurring from the change in dihedral angle conformation (panel **E**) occur with similar frequency as the vibrations in the conjugated double bond system of the hexatriene unit of previtamin D (panel **D**). In addition, the change in S_1 excitation energy (panel **A**) also leads to oscillations in the traces of the broadband spectrum, since the traces are taken at constant wavelengths, but the lambda-max values, which corresponds to the S_1 excitation energy, oscillate with similar frequency as the bond distances of the hexatriene unit.

We discuss this in the main manuscript, where we refer to the SI for a more detailed discussion:

> "Besides the discussed large-amplitude oscillations that are due to the change in the dihedral angle conformations, we notice that the entire TRCD spectrum exhibits

high-frequency oscillations with low amplitude. These high-frequency oscillations are due to high-frequency oscillations of the rotatory strengths, which we exemplify for an example trajectory in Figure S1 of the SI. Further analysis show, that the high-frequency oscillations of the rotatory strengths (Figure S1, panel **B**) with an approximate period of 20–30 fs match the oscillations of the bond distances conjugated double bond system of the central hexatriene unit of Pre (before bond breaking this is the cyclohexadiene unit of provitamin D) (Figure S1, **D**). Furthermore, also high-frequency oscillations in the S_1 excitation energies (Figure S1, **A**) introduce high-frequency oscillations in the traces of the broadband spectrum, since the traces are taken at constant wavelength, but the lambda-max values oscillates. These high-frequency oscillations are probably due to due density fluctuations caused by the bond vibrations of the central unit of the molecule. A more detailed analysis of the high-frequency, low-amplitude oscillations is found in the SI."

We added Figure S2 to the SI, which shows several parameters for an example trajectory that undergoes ring-opening. We discuss the Figure S2 in more detail in the SI:

"**Analysis of high-frequency oscillations**

To analyze the high-frequency, low-amplitude oscillations in the TRCD, we examine one example trajectory (Figure S2). The high-frequency oscillations in the TRCD spectrum are caused by high-frequency oscillations in the rotatory strength of the S_1 (panel **B**), which occurs with similar frequency as the bond vibrations in the central unit of the provitamin D/previtamin D (panel **D**). According to the dynamophore concept [10], the oscillations in cyclohexadiene unit are activated by the electronic transition to S_1, which eventually leads to the electrocyclic ring-opening to form the hexatriene derivative previtamin D.

Another reason for oscillations in the traces of the TRCD spectrum are the oscillations in the S_1 excitation energy (panel **A**). The maxima of absorption bands is given by the S_1 excitation energy; therefore the lambda-max values of the instantaneous spectra oscillate. However, since the traces of the broadband spectrum are taken at constant wavelengths, the oscillations in the excitation energies lead to an oscillation in the TRCD traces. This oscillation occurs with similar frequency as the bond oscillations of the central unit of provitamin D/previtamin D.

In summary, the high-frequency, low-amplitude oscillations are possibly due to the density fluctuations associated to the bond vibrations in the central double bond system of the molecule.

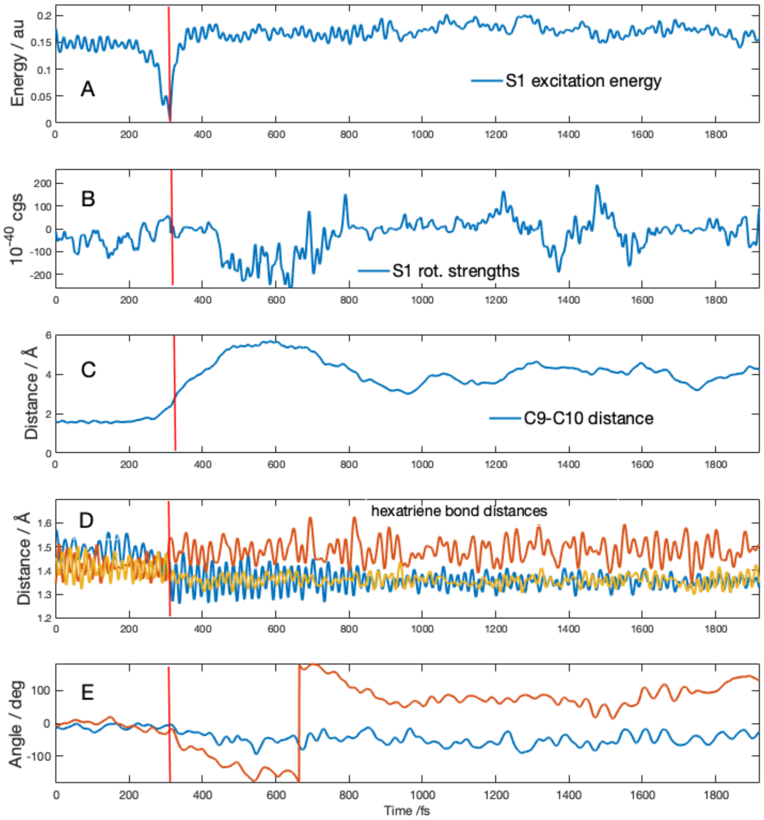

Figure 2: Time evolution of different parameters of an example ring-opening trajectory. The vertical line at 313 fs indicates the surface hop from S_1 to the ground state. **A**. Evolution of the S_1 excitation energy in au. **B**. Evolution of the rotatory strengths of S_1. **C**. Evolution of the bond-breaking coordinate (C9-C10 bond). **D**. Evolution of some of the bond distances in the hexatriene unit; blue: distance C9-C8, red: distance C8-C7, yellow: distance C7-C6. **E**. Evolution the dihedral angles ϕ_1 (red) and ϕ_2 (blue). Atom numbers are defined in Figure 1 of the main article."

Reviewer Comment:

5. It looks that many isomers are generated in the ground state after the internal conversion. Is it possible to give a Scheme to illustrate the whole mechanism, explaining the generation of all products and their correlation with time-resolved CD spectrum? This will largely help people to understand the whole mechanism.

Authors' response: In order to understand the mechanism better we added labels to Figure 4. However, since there is no uniform isomerization pathway it is difficult to summarize the isomerization process in a uniform scheme. We believe that Figure 4, left column summarize this mechanism as best as possible in all its complexity. The original publication (Tapavicza et al 2011) described this pathway with higher time resolution. Here, we focus on assigning the rotamers to the instantaneous CD spectrum, to assign the measured spectrum.

Reviewer Comment:

6. The simulation of the time-resolved spectrum is a very important topics in the on-the-fly nonadiabatic surface hopping dynamics field. More additional discussions on this field should be given and some important references should be mentioned.

Authors' response:

We added some important references, where non-adiabatic molecular dynamics simulations have been used to compute TR-spectra.

> "To obtain a relationship between the oscillatory structure of the TRCD, we apply non-adiabatic excited state molecular dynamics simulations [12, 14, 13], which have been shown to provide structural information of the photodynamics in a variety of organic systems[18, 9] and are therefore well-suited to complement pump-probe experiments [10, 7, 3, 15, 6, 19]."

References

[1] Rudiger Bauernschmitt and Reinhart Ahlrichs. Stability analysis for solutions of the closed shell kohn–sham equation. *J. Chem. Phys.*, 104(22):9047, June 1996.

[2] M. E. Casida. Time-dependent density functional response theory for molecules. In D. P. Chong, editor, *Recent Advances in Density Functional Methods*, pages 155–192. Singapore, World Scientific, 1995.

[3] Michael Filatov, Seunghoon Lee, Hiroya Nakata, and Cheol Ho Choi. Structural or population dynamics: What is revealed by the time-resolved photoelectron spectroscopy of 1, 3-cyclohexadiene? a study with an ensemble density functional theory method. *Phys. Chem. Chem. Phys.*, 22(31):17567–17573, 2020.

[4] F. Furche and R. Ahlrichs. Adiabatic time-dependent density functional methods for excited state properties. *J. Chem. Phys.*, 117:7433, 2002.

[5] Filipp Furche. On the density matrix based approach to time-dependent density functional response theory. *J. Chem. Phys.*, 114(14):5982–5992, 2001.

[6] Kevin Issler, Floriane Sturm, Jens Petersen, Marco Flock, Roland Mitrić, Ingo Fischer, Lou Barreau, and Lionel Poisson. Time-resolved photoelectron spectroscopy of 4-(dimethylamino)benzethyne – an experimental and computational study. *Phys. Chem. Chem. Phys.*, 25:9837–9845, 2023.

[7] Yusong Liu, Pratip Chakraborty, Spiridoula Matsika, and Thomas Weinacht. Excited state dynamics of cis, cis-1, 3-cyclooctadiene: Uv pump vuv probe time-resolved photoelectron spectroscopy. *J. Chem. Phys.*, 153(7):074301, 2020.

[8] Julia Meyer-Ilse, Denis Akimov, and Benjamin Dietzek. Ultrafast circular dichroism study of the ring opening of 7-dehydrocholesterol. *J. Phys. Chem. Lett.*, 3(2):182–185, 2012.

[9] Baswanth Oruganti, Péter Pál Kalapos, Varada Bhargav, Gábor London, and Bo Durbeej. Photoinduced changes in aromaticity facilitate electrocyclization of dithienylbenzene switches. *J. Am. Chem. Soc.*, 142(32):13941–13953, 2020.

[10] Oliver Schalk, Ting Geng, Travis Thompson, Noel Baluyot, Richard D. Thomas, Enrico Tapavicza, and Tony Hansson. Cyclohexadiene revisited: A time-resolved photoelectron spectroscopy and ab initio study. *J. Phys. Chem. A*, 120(15):2320–2329, 2016.

[11] Robert Send, Michael Kühn, and Filipp Furche. Assessing excited state methods by adiabatic excitation energies. *J. Chem. Theory Comput.*, 7(8):2376–2386, 2011.

[12] E. Tapavicza, I. Tavernelli, and U. Rothlisberger. Trajectory surface hopping within linear response time-dependent density-functional theory. *Phys. Rev. Lett.*, 98:023001, 2007.

[13] Enrico Tapavicza, Gregory D. Bellchambers, Jordan C. Vincent, and Filipp Furche. Ab initio non-adiabatic molecular dynamics. *Phys. Chem. Chem. Phys.*, 15:18336–18348, 2013.

[14] Enrico Tapavicza, Alexander M. Meyer, and Filipp Furche. Unravelling the details of vitamin D photosynthesis by non-adiabatic molecular dynamics simulations. *Phys. Chem. Chem. Phys.*, 13:20986, 2011.

[15] Chuncheng Wang, Max DJ Waters, Pengju Zhang, Jiří Suchan, Vít Svoboda, Tran Trung Luu, Conaill Perry, Zhong Yin, Petr Slavíček, and Hans Jakob Wörner. Different timescales during ultrafast stilbene isomerization in the gas and liquid phases revealed using time-resolved photoelectron spectroscopy. *Nat. Chem.*, 14(10):1126–1132, 2022.

[16] Ingolf Warnke, Sefer Ay, Stefan Bräse, and Filipp Furche. Chiral cooperativity and solvent-induced tautomerism effects in electronic circular dichroism spectra of [2.2]paracyclophane ketimines. *J. Phys. Chem. A*, 113(25):6987–6993, 2009.

[17] Ingolf Warnke and Filipp Furche. Circular dichroism: electronic. *Wiley Interdiscip. Rev. Comput. Mol. Sci.*, 2(1):150–166, 2012.

[18] Christian Wiebeler, Christina A. Bader, Cedrik Meier, and Stefan Schumacher. Optical spectrum, perceived color, refractive index, and non-adiabatic dynamics of the photochromic diarylethene cmte. *Phys. Chem. Chem. Phys.*, 2014.

[19] Monika Williams, Ruaridh Forbes, Hayley Weir, Kévin Veyrinas, Ryan J MacDonell, Andrey E Boguslavskiy, Michael S Schuurman, Albert Stolow, and Todd J Martinez. Unmasking the cis-stilbene phantom state via vacuum ultraviolet time-resolved photoelectron spectroscopy and ab initio multiple spawning. *J. Phys. Chem. Lett.*, 12(27):6363–6369, 2021.

jz-2023-008623.R2

Name: Peer Review Information for "Ab initio Simulation of the Ultrafast Circular Dichroism Spectrum of Provitamin D Ring-opening"

Second Round of Reviewer Comments

Reviewer: 2

Comments to the Author

The authors made the proper revisions to address my comments. Thus I recommended this work to JPCL

Author's Response to Peer Review Comments:

1. Description of Supporting Information has been added before the acknowledgement.

2. Page numbers of SI have been changed to S1, S2, ... etc.

3. Cover Art file has already been uploaded with the previous revision. Please let me know if you cannot find it or if there is anything wrong with file.
